# Decomposition of the drivers of the U.S. hospital spending growth, 2001–2009

**DOI:** 10.1186/1472-6963-14-230

**Published:** 2014-05-21

**Authors:** Vivian Y Wu, Yu-Chu Shen, Myeong-Su Yun, Glenn Melnick

**Affiliations:** 1 Sol Price School of Public Policy, University of Southern California, Los Angeles, CA, USA; 2 Graduate School of Business and Public Policy, Naval Postgraduate School, Monterey, CA, USA; 3 Department of Economics, Tulane University, New Orleans, LA, USA

**Keywords:** Hospital, Oaxaca-Blinder, Decomposition, Prices

## Abstract

**Background:**

United States health care spending rose rapidly in the 2000s, after a period of temporary slowdown in the 1990s. However, the description of the overall trend and the understanding of the underlying drivers of this trend are very limited. This study investigates how well historical hospital cost/revenue drivers explain the recent hospital spending trend in the 2000s, and how important each of these drivers is.

**Methods:**

We used aggregated time series data to describe the trend in total hospital spending, price, and quantity between 2001 and 2009. We used the Oaxaca-Blinder method to investigate the relative importance of major hospital cost/spending drivers (derived from the literature) in explaining the change in hospital spending patterns between 2001 and 2007. We assembled data from Medicare Cost Reports, American Hospital Association annual surveys, Prospective Payment System (PPS) Impact Files, Medicare Provider Analysis and Review (MedPAR) Medicare claims data, InterStudy reports, National Health Expenditure data, and Area Resource Files.

**Results:**

Aggregated time series trends show that high hospital spending between 2001 and 2009 appears to be driven by higher payment per unit of hospital output, not by increased utilization. Results using the Oaxaca-Blinder regression decomposition method indicate that changes in historically important spending drivers explain a limited 30% of unit-payment growth, but a higher 60% of utilization growth. Hospital staffing and labor-related costs, casemix, and demographics are the most important drivers of higher hospital revenue, utilization, and unit-payment. Technology is associated with lower utilization, higher unit payment, and limited increases in total revenue. Market competition, primarily because of increased managed care concentration, moderates total revenue growth by driving lower unit payment.

**Conclusions:**

Much of the rapidly rising hospital spending growth in the 2000s in the United States is driven by factors not commonly known or well measured. Future studies need to explore new factors and dynamics that drive longer-term hospital spending growth in recent years, particularly through the channel of higher prices.

## Background

U.S. healthcare spending accelerated considerably in the late 1990s and continued to grow rapidly until the recent recession. In particular, annual hospital spending growth was 4.1% and 8.7% in 2000 and 2001 respectively [1], and continued to increase at an average annual rate of eight percent between 2001 and 2007 [2]. The eight percent annual growth rate outpaced overall inflation and the growth of the economy during the same period. While overall healthcare spending slowed between 2008 and 2010, many believe that the slowdown is only temporary, caused by the most severe recession in the United States since the Great Depression [3,4]. Therefore, it is important to understand what drives the trend of rapid spending growth in the 2000s to inform the prediction of future spending growth following the economic recovery.

There are many studies which report the rising U.S. health care spending by year, over a short period of time, and/or in selected markets. However, only limited research has been done which systematically examines the potential drivers over a longer period of time, particularly for the most recent decade. Much of the related literature focused on the reversal of the hospital spending trend in the late 1990s through the early 2000s and provided inconsistent explanations. For example, Hay [5], using 1998–2001 data from a private health plan, showed that *economic characteristics, technology, and hospital market structure* are the most important factors explaining the (mostly between-state) variation in hospital cost. Another study covering the same period found that *Health Maintenance Organization (HMO) penetration* and caps on *malpractice* awards were the most important explanatory variables in hospital admissions/cost estimation [6]. A study from the Lewin Group [7], using data between 1998 and 2001 from three different sources, found that *provider market structure* and *physician supply* variables were most important in predicting hospital-based outpatient cost. Other studies analyzed hospital financial reports and provided descriptive evidence that rising costs for nurses and other personnel were one of the main causes of inpatient cost growth in the early 2000s [8-10]. In contrast, studies paid less attention to the continual spending growth after the early 2000s. Our research fills the gap in the literature by covering a longer period throughout the 2000s and using a more systematic approach to examine the underlying drivers of the spending pattern.

In this paper, we use data from all acute care hospitals in the United States from 2001 to 2009 to document hospital spending patterns under existing and evolving market conditions to guide future spending predictions. We then use data from 2001 to 2007 to conduct the Oaxaca-Blinder (OB) methodology, which decomposes per capita hospital spending growth into quantity and price trends, and assesses (a) how well the historically important hospital cost/spending drivers together explain the sustained spending growth in the most recent decade, and (b) how important these factors each are. Oaxaca [11] and Blinder [12] first introduced the method to decompose the differences in outcomes between groups into two broad categories: (a) change in outcomes due to the change in the values of the observed characteristics, and (b) change in outcomes due to changes in the relationship between outcomes and observed characteristics. This methodology enables us to understand how change in hospital spending over time is explained by the change in the share of for-profit (FP) hospitals, for example, versus explained by the changing relationship FP ownership has on hospital spending. As such, the information in this paper will help policy makers identify critical individual factors that contribute to the spending growth and devise policies to moderate the driving forces. The OB methodology has been extended to a large volume of health economic and health outcomes studies. For example, the OB method is used extensively to decompose the disparity in health care access and use between different race/ethnic groups [13,14], changes in insurance coverage over time [15,16], and the obesity rise in recent decades [17]. In this study, we apply the model to examine the rise in hospital spending between 2001 and 2007. Because the hospital sector is the largest provider of health care in the United States—whose spending comprises about one third of total health care expenditures—we focus on analyzing the drivers of hospital spending.

## Methods

### Data source

Our main analytical data file [1] is compiled from several data sources. The data on hospital net patient revenue and hospital labor salary and wage are drawn from Medicare hospital cost reports, 2001 to 2009. Hospital utilization data, organizational characteristics, technology availability, and staffing information are from the American Hospital Association (AHA) annual surveys. The Medicare casemix index is obtained from the Medicare Hospital Inpatient PPS Impact Files. We used data from MedPAR Medicare claims between 2001 and 2005 to calculate the number of acute myocardial infarction (AMI) patients and their percutaneous transluminal coronary angioplasty (PTCA) or coronary artery bypass graft (CABG) treatments. The managed care data for years 2001 and 2004 are compiled from HMO and Preferred Provider Organization (PPO) enrollment data from InterStudy. Hospital concentration measures are provided by Dr. Glenn Melnick [18]. Measures on percent revenue by Medicare and Medicaid are derived from National Health Care Expenditure data from Center of Medicare and Medicaid Services, 2001–2005. Lastly, county characteristics such as per capita income, unemployment rate, and population structure are obtained from the Area Resource File. The aggregated time series sample includes all general, acute, non-federal hospitals located in Metropolitan Statistical Areas (MSAs) that continuously operated between 2001 and 2009. The decomposition sample is restricted to similar hospitals that operated continuously between 2001 and 2007, excluding outliers (i.e., hospitals whose reported values of the three main outcome measure—net patient revenue, adjusted days, and average payment per adjusted day—are in the top and bottom one percent of the distribution in 2001 or 2007).

### Statistical model

The study design combines the analysis of aggregate time-series data 2001–2009 and OB regression-based decomposition methods [11,12,19] to identify the factors that explain hospital revenue growth between 2001 and 2007. The decomposition analysis is limited to 2001 to 2007 just prior to the recent recession because spending and utilization patterns might be different under economic contraction, and our managed care data is available only up to 2004. Considering the differences in hospital net revenue between 2001 and 2007:

(1) $${\bar{Y}}_{2007}-{\bar{Y}}_{2001}={\bar{X}}_{2007}^{\prime }\beta_{2007}+{\bar{X}}_{2001}^{\prime }\beta_{2001}$$

Equation 1 shows the change in hospital revenue (Y) in 2007 and 2001, evaluated at the yearly value of the independent variables (X) and their corresponding effect on hospital revenue (the coefficient, β). After simple manipulation, we obtained the following relationship:

(2) $$\begin{array}{l} {\bar{Y}}_{2007}-{\bar{Y}}_{2001}=\left({\bar{X}}_{2007}^{\prime }-{\bar{X}}_{2001}^{\prime }\right)\;\beta_{2001}\\ \;+{\bar{X}}_{2007}^{\prime }\left(\beta_{2007}-\beta_{2001}\right) \end{array}$$

The observed growth in hospital revenue thus can be decomposed into two components: the first part of Equation 2 represents the portion of hospital revenue growth that can be explained by changes in the values of the observed characteristics; the second part of Equation 2 represents the portion of revenue growth caused by changes in how these observed factors affect a hospital’s revenue over this period (changes in coefficients) and other factors not captured in the model. The focus of this analysis is on decomposing the first part of Equation 2, or explaining the contribution of different categories of observed characteristics to hospital revenue growth.

We chose 2001’s coefficients as the base for the decomposition, because we are interested in examining how hospital spending patterns have changed since 2001.

### Outcome variables

To investigate the underlying mechanism driving hospital inpatient spending growth between 2001 and 2007, we analyzed growth rates in three ways: the hospital’s net patient revenue, patient volume, and average unit payment. The hospital’s net patient revenue is measured as gross patient revenue minus contractual allowances and bad debts. We used real hospital net patient revenue by deflating nominal net patient revenue with consumer price index. The hospital’s patient volume is measured by adjusted days: Adjusted days are calculated as total actual inpatient days plus estimated equivalent inpatient days related to outpatient services volume using the AHA methodology^a^. Lastly, unit payment is measured as net patient revenue per adjusted days. For the first two outcome measures, we used the log transformation in the analyses to account for the highly skewed distribution of hospital revenue and output. Examining the three outcomes enabled us to see whether the growth is driven mainly by quantity or by unit price. We then applied the OB decomposition to each of the three outcomes separately.

### Key explanatory variables

Building on past literature, hospital cost/revenue is assumed to be a function of many determinants, including advances in medical technology and patient, hospital, and payer factors [20]. In our present study, we are most interested in three categories of variables that the literature suggests as the main causes of the late 1990 to early 2000 resurgence in hospital spending: (a) labor cost, (b) technology, and (c) market competition. First, rising labor cost is critical to the study time period, given the wide media coverage of nursing and healthcare staff shortage since late 1990 [6], and several studies have provided some evidence on this cause [8-10]. Second, advances in new medical technology have been identified as the key determinant of long-term health care/hospital expenditure growth [21-24]. Other studies have documented a boom in the use of imaging equipment since the early 2000s [25,26] and an expansion in cardiac services [27], suggesting the importance of new technology and its potential effect on hospital spending during the study period. Third, hospitals continue to consolidate into systems, and several studies indicate that hospital consolidations are associated with higher hospital prices in recent years [28-30]. Other studies indicate disenrollment from HMOs to less restrictive PPO plans and the HMOs easing restrictions on access to and utilization of health care services as the explanation for increased spending [31]. Literature also suggests that additional factors such as hospital ownership, aging of the population (particularly the baby boomers [24]), and increased economic conditions may also be important in driving higher hospital spending. We include a comprehensive list of variables and group them into the following categories:

• *Hospital Staffing Level and Labor-Related Costs*. We include (a) a measure of total labor-related costs per hour, which sums up a hospital’s total employees’ salaries, wages, and other labor costs such as contracted nurses, therapists, management, pharmacy, laboratories, and pension and benefit costs, and (b) total full-time equivalent (FTE) personnel per bed, (c) FTE registered nurses per bed, (d) FTE licensed practitioners per bed, and (e) FTE resident doctors per bed.

• *Technology*. We considered technologies that are considered high-tech, profitable services [32] and are consistently recorded in AHA surveys during the study period. For cardiac services, we indicate whether a hospital has (a) a cardiac catheterization lab or (b) an open-heart surgery facility. To additionally account for the intensity of use, we controlled (c) number of Medicare AMI patients treated in a hospital, (d) percent of Medicare AMI patients receiving PTCA, and (e) percent of Medicare AMI patients receiving CABG surgery. Other high-tech equipment and services included are (f) positron emission tomography (PET), (g) single photo emission computerized tomography (SPET), (h) extracorporeal shock wave lithotripsy, (i) magnetic resonance imaging (MRI), and (j) neonatal intensive care.

• *Market Competition.* Three key measures of market competition variables are included: (a) managed care (MC) penetration, (b) the MC Herfindahl-Hirschman index (HHI), and (c) the hospital HHI. The insurer-hospital bargaining literature suggests that both the insurer and the hospital side of market structure are important in determining the utilization and payment of hospital services [33]. Insurer market structure is measured by MC penetration and MC HHI at the MSA level. We used a combined HMO and PPO measure following the conceptual argument that HMO and PPO may be substitutes so that they should be considered in the same relevant product market [34,35]. Hospital-specific HHI’s are derived from Medicare discharge MedPAR data using actual zip code level patient flow data, following the detailed method described in Bamezai et al. [21].

• *Hospital Organizational Characteristics*: A hospital’s ownership and organizational structure will affect its mission and value for profit, which in turn will affect cost and pricing behavior. For this category, we include a hospital’s (a) ownership status (for-profit, government, and not-for-profit), (b) whether a hospital is a teaching hospital, (c) number of available beds (in seven categories of bed sizes), (d) hospital occupancy rate, indication of whether or not there is excess capacity [36,37], and (e) whether a hospital is part of a hospital system.

• *Casemix and Socio-Economic Demographics*: One potential driver of hospital spending is an increasingly sick and aging patient population. We used (a) the Medicare casemix index to control the general severity of illness of the patient population (with the assumption that sicker patients are more costly to treat). We also included local demand-side socio-economic characteristics and physician supply, which include (b) per capita income at the county level, (c) unemployment rate, (d) percent of elderly population, and (e) number of physicians per capita.

• *Hospital Payer Mix*: A hospital’s total net patient revenue depends on the mix of revenues from different payers. Revenues from private payers can be affected by payment generosity of public payers. Therefore, we included a payer-mix variable for Medicare and Medicaid each, which calculates the percent of total hospital revenue that is paid for by Medicare and Medicaid at the state level, respectively.

## Results

**Figure 1:**
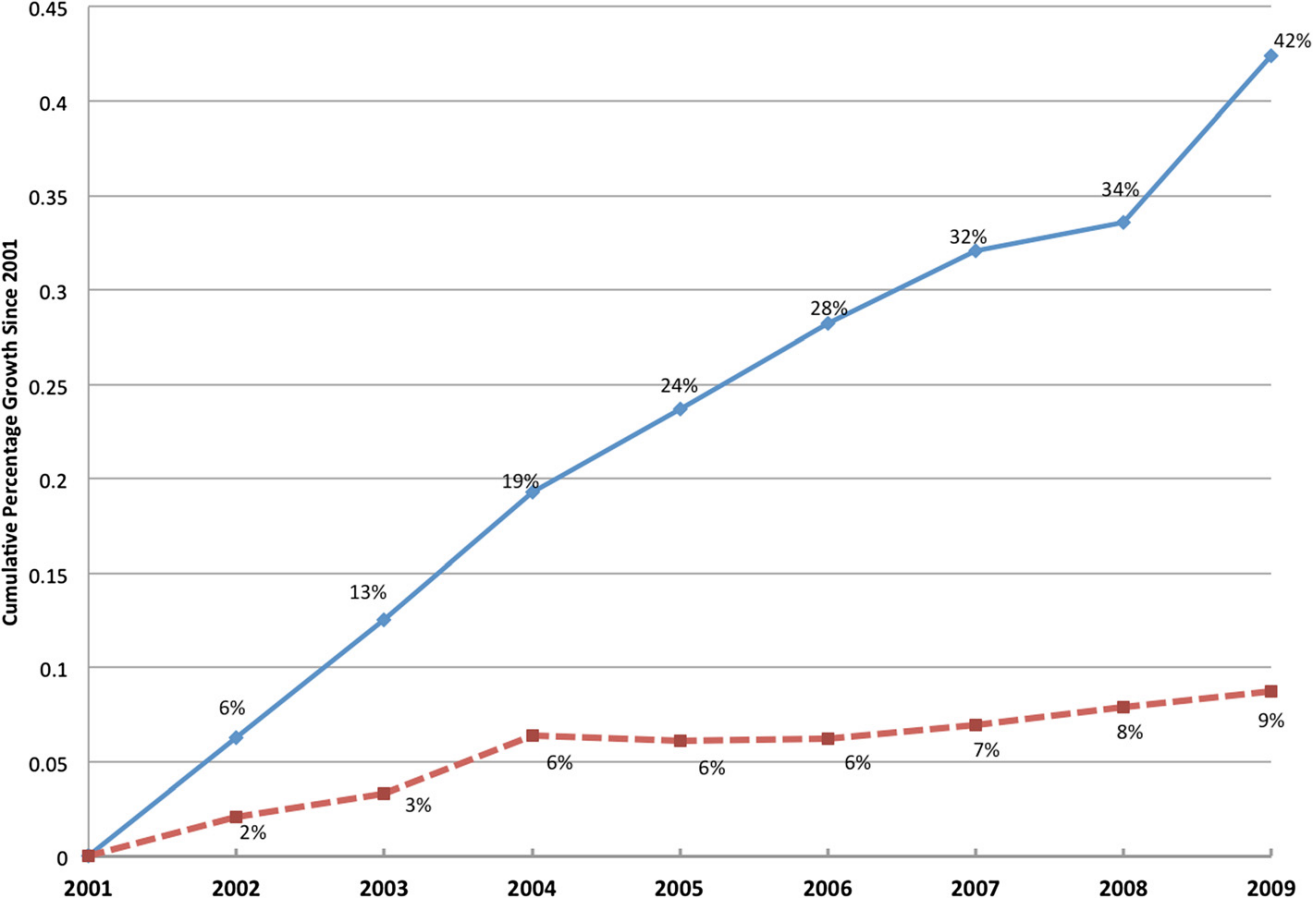
**Cumulative Growth in Real Hospital Net Revenue and Quantity, 2001–2009.** - (solid line, upper) Real Hospital Net Revenue per capita, - (dash line, lower) Quantity-Adjusted Patient Days per capita.

Figure 1 presents the aggregated time series of overall trend data for total hospital spending (average net patient revenue) per capita and utilization (adjusted patient days) per capita between 2001 and 2009. With the weakening of managed care since the late 1990s, one would expect hospital utilization to grow quickly. However, utilization appears to account for a very limited portion of the total growth in net patient revenue. During 2001 to 2009, nominal and real (deflated by consumer price index) total net patient revenue grew by 64% and 42%, respectively, while utilization grew by a total of nine percent only. This trend implies that much of the large revenue growth is caused by more expensive payment per unit of adjusted hospital day, and that these trends are more than a short-term phenomenon.

Table 1 provides summary statistics of the potential drivers of hospital spending that are used in the decomposition analysis. This table shows that the largest changes between 2001 and 2007 occurred in the staffing and labor costs, technology expansion, and market competition categories. The number of FTEs per bed has increased, mainly because of an increase (25%) in RNs per bed. The hospital’s average labor-related cost rose by 16%. PET and PTCA have expanded significantly: the number of hospitals owning PET increased by 35%, and the number of Medicare AMI patients receiving PTCA rose by 27%. The insurance market structure has changed as well: total managed care penetration (combined HMO and PPO penetration) declined by 12%, while the market’s concentration increased by 11%. Hospital market concentration has remained relatively stable. Other important changes are that share of FP hospitals increased by four percent, and that hospitals have either become small (hospitals with fewer than 100 beds grew by 11%) or very large (hospitals with more than 500 beds grew by 20%).

**Table 1:** Characteristics of hospitals and hospital environments in 2001 and 2007

	**2001**	**2007**	**Change**
Net patient revenue (millions)	$126	$193	53%
Adjusted days (thousands)	89	98	10%
Net patient revenue per adjusted day	$1,377	$1,961	42%
Staffing and labor-related costs			
Labor-related cost per hour	88.9	103.3	16%
FTE per bed	4.8	5.5	14%
RN per bed	1.2	1.5	25%
LPN per bed	0.2	0.1	-10%
Residents per bed	0.1	0.1	5%
Technology			
% Hospitals with cardiac catherterization lab	67%	65%	-3%
% Hospitals with open heart surgery facility	41%	45%	10%
# of Medicare AMI cases	66.8	55.3	-17%
% Medicare AMI receiving PTCA	12%	15%	27%
% Medicare AMI receiving CABG	4%	3%	-10%
% Hospitals with PET	17%	23%	35%
% Hospitals with SPECT	61%	58%	-6%
% Hospitals with ESWL	34%	37%	9%
% Hospitals with MRI	78%	82%	4%
% Hospitals with NICU	34%	37%	7%
Market competition			
MC penetration	64%	57%	-12%
MC HHI	1600	1769	11%
Hospital HHI	3511	3454	-2%
Hospital Characteristics			
% FP Hospital	18%	19%	4%
% Government	12%	12%	1%
% Teaching	10%	9%	-6%
% Hospital with < =100 beds	17%	19%	11%
% Hospital with 101–150 beds	16%	15%	-8%
% Hospital with 151–200 beds	14%	14%	-6%
% Hospital with 201–300 beds	22%	22%	0%
% Hospital with 301–500 beds	23%	22%	-5%
% Hospital with >500 beds	7%	9%	20%
Occupancy Rate	66%	66%	-1%
Member of a hospital system	60%	63%	6%
Casemix and demographics			
Medicare casemix	1.41	1.45	3%
Per capita Income	$30,070	$36,711	22%
Unemployment rate	5%	4%	-11%
% population > 65 y/o	12%	12%	-2%
MD per capita	2.76	2.81	2%
Payer Mix			
% Revenue medicare	31%	30%	-5%
% Revenue medicaid	17%	17%	-5%
Observations	1,704	1,642	

We then applied the OB method to examine the sources of growth in total net patient revenue and its volume and price components. Table 2 presents the decomposition results. The first row shows the total actual differences in the dependent variable values between 2001 and 2007. For example, the difference in log of net patient revenue is 0.41, meaning that the average growth rate in net patient revenue is 41% between 2001 and 2007. The next two rows separate the total difference into the percentage that can be attributed to change in the observed factors over time (explained) and percentage are due to coefficient effect and other unobserved factors (unexplained). The bottom half of Table 2 breaks down the total explained difference into its component parts: staffing and labor-related costs, technology, market competition, hospital characteristics, casemix and socio-economic demographics, and payer mix.

**Table 2:** Regression-based decomposition of real hospital net revenue growth between 2001 and 2007

	**Revenue**		**Utilization**		**Unit-Payment**	
	**Log of real hospital net revenue**		**Log of adjusted days**		**Real hospital net revenue per adjusted day**	
**Overall**						
2001	$18.31		11.14		$1,378	
2007	$18.72		11.21		$1,965	
**Difference**^**+**^	41	100%	7	100%	$587	100%
Explained	18	44%	4	57%	$173	30%
Unexplained	23	56%	3	43%	$413	70%
**Decomposition**						
Explained	18	100%	4.0	100%	$173	100%
Staffing and labor-related costs	12.1^**^	67%	3.3^**^	83%	$108^**^	62%
Technology	0.1	1%	-0.8^**^	-20%	$17^**^	10%
Market competition	-0.8^**^	-4%	0.2	5%	-$16^**^	-9%%
Hospital char	0.8^**^	4%	0.5^*^	13%	$2	1%
Casemix and demographics	4.8^**^	27%	0.6	15%	$52^**^	30%
Payer mix	0.8^**^	4%	0.4	10%	$9^**^	5%

^+^Expressed in percentage points except for the column of unit-payment.

^**^Indicates the predicted growth for that category is statistically significant at .01 level and * at .05 level.

The results show that the variables included in the regression can together explain 44% of the growth in net patient revenue, 57% of the growth in adjusted days, and 30% of the growth in price. The growth in net patient revenue is primarily driven by higher staffing and labor-related costs. The changes in staffing and labor-related hourly cost between 2001 and 2007 alone would predict a 12% increase in net patient revenue, accounting for 67% of the total growth that can be explained by all the observables. The second major driver is the change in casemix and demographics variables, accounting for 27% of the explained growth. Market competition, hospital characteristics, and payer mix categories are equally important as the third important driver. It is important to note that changes in market competition actually lowered total revenue growth. Without the observed change in market conditions, net patient revenue would have increased 0.8 percent.

Hospital utilization (adjusted patient days) grew by a much smaller seven percent since 2001, and the observed variables explain a large (57%) portion of the growth. The top two categories of drivers of higher utilization are staffing and labor-related costs (83%) and casemix and demographic factors (15%). Note that Table 1 showed a net increase in the availability of hospital technology we measured between 2001 and 2007. Therefore, the expansion of technology contributed to *lower* hospital utilization (-20%). Change in market competition played a very limited role in explaining utilization growth.

As described earlier, payment (measured by net patient revenue per adjusted days) growth drives most of the total net revenue increase in the study period. However, changes in the value of the observed variables could only explain 30% of the payment growth. Similar to the previous two outcomes we examined, staffing and labor-related costs is again the leading cause, accounting for 62% of the explainable payment growth. The second important driver is the casemix and demographic category (30%). More technology has led to a small increase in unit payment growth. Market competition moderated the unit payment growth, contributing to minus nine percent of the explained growth.

## Discussion

Our aggregated time series results show that the rapid growth in hospital spending between 2001 and 2009 is mostly driven by higher payment (per adjusted hospital day), not utilization. With regression-based decomposition, we found that changes in the value of the comprehensive set of variables can explain less than half of the total spending growth in 2001–2007. Two categories, staffing and labor-related costs and casemix and socio-economic demographics are the leading factors consistently driving higher total net patient revenue, utilization, and unit payment between 2001 and 2007. This result is consistent with several studies that document a nursing shortage [38,39] as well as a potential emerging (primary care) physician shortage [40,41]. However, while much of the utilization growth is explained, most of the more significant growth in unit payment is left unexplained. This finding is consistent with some recent studies which analyzed local hospital spending patterns, one of which found that higher hospital price growth in California is not related to changes in market structure or other factors in the model [42], and another that found that in Massachusetts, large variation in hospital prices is much better explained by hospital market power than by patient diagnosis/severity or hospital quality [43]. Our finding furthers the evidence that increasing unit payment for hospital services is a national trend, even if we cannot clearly explain this national trend based on existing measures.

Technology plays a mixed role in driving total hospital spending in the study period. On the one hand, our results show that the expansion of high-tech hospital services reduces the need of more intensive, higher-cost inpatient care. On the other hand, new technology is contributing to higher unit payment. The net effect is that technology is associated with a more limited growth in total hospital spending. While our technology measure captured most important new capital equipment hospitals adopted in the 2000’s, there are other potential omitted variables. For example, we do not capture the use of beta-blocker, new type of stents, and other medications and devices that also diffused widely throughout the 2000’s. Because we do not know how our measured capital equipment and these other omitted treatment technology are correlated, our estimated importance of technology can be biased in either direction.

Contrary to conventional thought that the retreat of HMO plans may have led to increased utilization and therefore hospital spending, we did not find evidence to support this claim. Total utilization growth in 2001 to 2007 is limited, and market competition variables including MC penetration, MC concentration, and hospital concentration do not explain much of this growth. It is possible that while managed care plans have relaxed the traditional gate-keeping and utilization authorizations/reviews, newer tools such as case and disease management strategies may be holding the utilization growth in control. However, we have no information on the new management strategies to directly test this hypothesis.

Our finding that market competition is associated with lower hospital payment between 2001 and 2007 is primarily driven by the increased managed care concentration during this period. During the study period, MC penetration decreased by about the same amount (12%) as the increase in MC concentration (11%), while hospital market structure remained relatively unchanged. Our findings show that increased MC concentration can suppress payment growth, suggesting a critical role of concentrated health plans in lowering payment in the 2000s. This is consistent with a recent study that found that increased concentration in health plan markets is associated with lower hospital price [44]. However, such dampening effect is offset by the growth in labor costs and aging population that increases overall spending.

It is possible that our measure of price—net patient revenue per day—is rising simply because of decreasing average length of stay (LOS) within an admission. We examined the effect that changing LOS might have on our results using hospital admission as an alternative unit of volume. In the sensitivity test, we found that LOS is fairly stable over time, and re-estimating Figure 1 using admissions as the unit of volume provided a very similar trend. Repeating the decomposition analysis adding LOS as an additional variable showed that LOS explained a small portion (4%) of the unit-payment growth in 2001–2007. Therefore, we ruled out the possibility that the trend in rising price per day is driven by differential trends in LOS and admissions.

Several limitations may affect the interpretation of our findings. First, study sample is limited to urban hospitals because we only have insurer market information on metropolitan areas. Therefore, our results may not apply to rural hospitals. Second, our data contain overall revenue reduction from billed charges as an estimate of net revenue hospital received. While this information is well suited to examine overall trends, it is not sufficient to investigate more detailed mechanisms as to which department or what type of services are contributing to the rising revenue/price. Third, while the OB method is a sound methodology to decompose changes in hospital spending into contributions by individual factors, it does not estimate a causal relationship. Therefore, our findings should be considered as an association between hospital spending and its contributing factors rather than causality.

## Conclusions

Overall, our results document a new and important trend of higher hospital spending, which appears to be driven by rapidly rising hospital prices (higher inpatient payment per adjusted day). In addition, factors that were commonly believed to drive hospital cost/price do not seem to explain much of the price growth. Future research is needed to understand and measure varied new activities occurring in the health plans and hospital markets that contribute to the high and rising provider prices in the last decade. Higher hospital prices are also likely caused by changing effects of market concentration related to the wave of provider market consolidation in the 2000s, and thus greater attention and efforts are also needed in both the public and private sectors to restore price competition. Antitrust agencies might need to move more aggressively to protect and restore market competition. Private sector innovation is needed to increase consumer price shopping both in the insurance markets and for medical care services.

### Endnotes

^a^Specifically, adjusted patient days = inpatient days + [inpatient days* (outpatient revenue/inpatient revenue)].

## Competing interests

The authors declare that they have no competing interests.

## Authors’ contributions

VYW conceived of the study, performed the statistical analysis, and drafted the manuscript. YS participated in the study design, helped constructing the analytical data files, and YS and GM helped the interpretation of the results and revision of the manuscript. MY provided conceptual and methodological application of the OB decomposition method, and helped the interpretation of the results and revision of the manuscript. All authors read and approved the final manuscript.

## Pre-publication history

The pre-publication history for this paper can be accessed here:

http://www.biomedcentral.com/1472-6963/14/230/prepub
